# Exploring the relationship between physical activity level and work-related demographic factors among emergency and outpatient nurses: a cross-sectional correlational study

**DOI:** 10.1186/s12912-025-03792-5

**Published:** 2025-09-02

**Authors:** Nita Fitria, Putri Karisa, Geraldino Hendy Manseaur, Silvya Dwi Yanti, Ajeng Fitrianingrum, Nadila Ramdhiani, Dewi Purwati, Muhamad Gustaf Al Fajar

**Affiliations:** 1 Department of Fundamental Nursing, Faculty of Nursing, Universitas Padjadjaran, Jl. Raya Bandung-Sumedang, KM. 21, Jatinangor, Sumedang, West Java Indonesia; 2 Biomedical Science, Faculty of Medicine, Universitas Padjadjaran, Bandung, West Java Indonesia; 3 Faculty of Nursing, Universitas Padjadjaran, Sumedang, West Java Indonesia; 4 Nursing Department, University of Indonesia Teaching Hospital, Depok, West Java Indonesia

**Keywords:** Cross-sectional study, Emergency nurses, Occupational health, Outpatient nurses, Physical activity

## Abstract

**Background:**

Physical activity plays an important role in maintaining the health and performance of nurses, especially those working in outpatient room (OR) and emergency room (ER) that has a high workload. However, data on the level of physical activity and its influencing factors among these nurses is unclear.

**Purpose:**

This study aimed to determine and compare the physical activity levels of ER and OR nurses and the relationship between physical activity levels and work-related demographic factors (age, length of work, unit room/department, gender, and career level).

**Patients and methods:**

Our study used a correlational design with 117 nurses (57 ER and 60 OR nurses). This study used the Global Physical Activity Questionnaire (GPAQ) as the instrument. Data were analyzed with the Spearman test.

**Results:**

A total of 117 nurses completed the survey. Most participants reported high physical activity level (*n* = 77, 65.8%), low (*n* = 24, 20.5%) and moderate (*n* = 16, 13,7%). Furthermore, the study found a significant weak positive correlation between physical activity with age (*r* = 0.25, *p* = 0.006) and length of work (*r* = 0.24, *p* = 0.010). But no significant correlation were found with gender, career level and unit room (*p* > 0.05).

**Conclusion:**

The findings suggest that age and length of work are significantly associated with physical activity levels among emergency and outpatient nurses. These results highlight the need for age- and experience-specific strategies to encourage and maintain physical activity in the nursing workforce.

## Introduction

Physical activity is widely recognized as a fundamental component of overall health and well-being [1]. It contributes to the prevention of chronic diseases, enhances mental health, and improves quality of life [2]. For healthcare professionals such as nurses, who are frequently exposed to physical and emotional stress, maintaining an adequate level of physical activity is not only essential for their own health, but also crucial for their ability to provide safe and effective patient care [3]. Ironically, despite being advocates for healthy lifestyles, nurses often struggle to engage in regular physical activity due to demanding work schedules, fatigue, and environmental constraints within the healthcare setting [4, 5].

Nursing roles vary considerably depending on clinical settings, and these differences may impact their physical activity levels [6]. Emergency Room (ER) nurses often face high-acuity patients, fast-paced decision-making, and unpredictable workloads that can lead to both physical exhaustion and psychological strain [7]. In contrast, Outpatient Room (OR) nurses may experience more consistent routines, different patient interactions, and potentially less physical strain [8]. These contrasting work environments could lead to significant differences in activity levels and related influencing factors [6]. However, limited research has directly compared physical activity patterns between nurses in emergency and outpatient departments, particularly in relation to occupational and socio-demographic factors such as workload in the room, age, length of work, gender, and carrier level [6, 9].

Different with previous research focusing on single settings or general nurse populations, this study provides a unit-specific comparison with actionable insight for tailored interventions. These two groups were selected due to their contrasting work characteristics: emergency nurses face irregular shifts, intense physical demands, and high-pressure environments, which can both enhance or hinder physical activity depending on fatigue levels [10–12]. Outpatient nurses, by contrast, typically work stable day shifts with lower physical demands and potentially more consistent opportunities for leisure-time activity [13, 14]. Compared to other units like ICU or inpatient wards, emergency nurses are more physically active yet more strained, while ICU nurses, though under high psychological stress, may have limited movement due to patient monitoring tasks [15, 16]. This comparison provides insight into how sociodemographic factors shape physical activity patterns and supports the need for unit-specific health promotion strategies [17].

Understanding how work environments influence physical activity among nurses is vital, not only from a health promotion standpoint but also in terms of workforce sustainability and patient care outcomes. Notably, studies suggest the temporal impact of work characteristics is not a problem in nursing because it is difficult to avoid, especially in emergency conditions such as COVID-19 [18–20]. These problems cause nurses’ opportunities for physical activity to decrease [21]. A meta-analysis conducted by Khani et al. showed that over the past two decades, sedentary lifestyles and lack of physical activity have been the main factors for various diseases (such as cardiovascular disease and diabetes mellitus) to occur in nurses [22]. Identifying specific work-related determinants of physical activity can guide targeted strategies to support the health of nursing staff, especially in high-demand clinical settings.

Despite growing awareness of occupational health in nursing, few studies have directly compared physical activity levels between ER and OR nurses, and none have comprehensively examined how work-related demographic factors in these settings. This gap is significant, as inadequate physical activity among nurses is associated with musculoskeletal disorders, cardiovascular risk, and decreased work performance [23]. This study aimed to assess and compare physical activity levels between emergency and outpatient nurses and to examine their associations with work-related demographic factors (age, length of work, unit/department, gender, and career level). The research was guided by two questions: [1] What are the physical activity levels among emergency and outpatient nurses? and [2] How are these levels associated with work-related demographic factors?.

## Materials and methods

### Study design

A cross-sectional correlational quantitative study approach was conducted from July to August 2023.

### Sample/participants/informant

This study was conducted in the nursing unit of a hospital in Bandung, Indonesia. The study population consisted of Emergency Room (ER) and Outpatient Room (OR) nurses. Inclusion criteria were: (a) being a nurse with at least one year of work experience, (b) working in the emergency and outpatient units, (c) voluntarily agreeing to participate in the study and signing the informed consent, and (d) not being on leave or work permit during the past one month. A total of 117 nurses from the emergency and outpatient departments participated in this study, with no refusals or dropouts. The sampling technique used was purposive sampling method, selecting participants based on specific inclusion criteria relevant to the study objectives. The required sample size was calculated using G*Power software with an assumed effect size of 0.5, power of 80%, and significance level of 5%, resulting in a minimum of 102 participants. Thus, the final sample size was deemed adequate.

### Instrument

Physical activity levels were assessed using the Global Physical Activity Questionnaire (GPAQ), developed by the World Health Organization (WHO) [24]. The GPAQ measures physical activity according to the minute Metabolic Equivalent of Task (MET), which is a valid and reliable standardized metric for estimating energy expenditure during physical activity relative to resting metabolic rate. MET is measured by the following formula:

$$\begin{aligned}\:&Weekly\:exercise\:score\:\\&=\:\left(0\:*\:times\:of\:light\:exercise\:per\:week\right)\:\\&+\:\left(4\:*\:times\:of\:moderate\:exercise\:per\:week\right)\\&\:+\:\left(8\:*\:times\:of\:heavy\:exercise\:per\:week\right).\end{aligned}$$

This instrument is a standardized instrument adopted from Istiqamah et al. (2021) and has been translated into Indonesian, with a validity and reliability value of Cronbach’s alpha 0.89, demonstrating good stability over repeat administrations [25]. It consists of 16 items with three domains: activities at work (6 items), traveling to and from a place (3 items), and leisure activities (7 items). The questionnaire is multidimensional allowing it to describe participants’ physical activity profile in the context of work, leisure, household, and transportation. Exploring the frequency and duration of engagement in physical activity for at least 10 min during the previous week allowed for more comprehensive information. Based on the GPAQ analysis guidelines, participants were categorized into low, moderate, or high physical activity levels according to their reported MET-minutes per week, considering frequency, duration, and intensity across work, transport, and leisure domains. Physical activity levels were classified according to MET minutes calculated per week, including [1] if the participant spent < 600 MET minutes, low activity was defined, [2] 600-2,999 MET minutes for moderate activity, and ≥ 3,000 MET minutes for high activity [26, 27].

### Data collection

Data was collected between July and August 2023 through an online self-administered questionnaire designed by the investigators. The questionnaire consisted of: [1] information about the study, instructions for completion, and informed consent, [2] demographic characteristics, and [3] General Physical Activity Questionnaire (GPAQ). Eligible subjects were contacted, and collected in one place. The questionnaire was distributed via google form with an estimated duration of 15 min. Completion was done simultaneously at the same location.

### Data analysis

Data analysis is presented in the form of frequency and percentage distribution tables. This study analyzed data using univariate and bivariate methods. Descriptive statistics using frequency distribution and percentages were used to summarize the characteristics of the participants as a univariate analysis. This study has an abnormal data distribution, a bivariate analysis was conducted using the Spearman Rank Test with a 95% significance level and effect size (rho) to quantify correlation strength. IBM SPSS software version 27 was used to analyzed the data.

### Ethical consideration

This study was approved by the Medical Ethics and Research Council at Universitas Padjadjaran (32/UN6.KEP/EC/2023). In addition, permission to use the instrument was requested from the authors of the original translated version of the GPAQ. The research permit was also approved by the Hospital Ethics Committee and consent to participate was obtained from all participants in this study. Participants were informed about the purpose of the study, as well as the confidential and anonymized nature of the data. All participants signed a consent form prior to participation. The Helsinki Declaration guidelines were followed in this study.

## Results

### Characteristics of participants

The characteristics of participants in this study are presented in Table 1 (Table 1). Table 1 shows that it is known that out of 117 participants, most were OR nurses (51.3%). Almost half of the participants were in the 36–45 year age group (47%) and had work experience of ≥ 22 years (38.5%). Most participants were at the nurse supervisor career level (57.3%). Based on gender, it is known that most participants are female, 82 (70.1%), rather than male, 35 (29.9%).

**Table 1:** Characteristics of participants according to the demographic data (*n* = 117)

Characteristics	Frequency (f)	Percentage (%)
**Gender**		
Male	35	29.9
Female	82	70.1
**Unit Room**		
Emergency room	57	48.7%
Outpatient room	60	51.3%
**Age**		
26–35 years old	22	18.8%
36–45 years old	55	47%
46–55 years old	28	23.9%
56–65 years old	12	10.3%
**Lengths of Works**		
1–3 years	8	6.8%
4–9 years	11	9.4%
10–18 years	34	29.1%
18–21 years	19	16.2%
≥ 22 years	45	38.5%
**Career Level**		
Registered Nurse	16	13.7%
Experienced Registered Nurse	33	28.2%
Nurse Supervisor	67	57.3%
Nurse Manager	1	0.8%

### Physical activity level

Among the 117 study participants, 65.8% were involved in high, 13.7% in moderate and 20.5% in low physical activity level (Table 2). In the correlational analysis, we assessed the relationship between physical activity level and participants’ characteristics, including gender, room, age, length of works and career level. It was found that nurses’ physical activity level had a weak positive correlation with age (*r* = 0.25; *p* = 0.006) and lengths of work (*r* = 0.24; *p* = 0.010). No significant correlation were found with gender, unit room and career level (Table 3). Although the correlations between physical activity and both age and length of work were statistically significant, their strengths were weak. However, these weak positive correlation may reflect practical dynamics in the workplace. In some clinical settings, senior nurses with more experience are often entrusted with greater responsibilities and may be more actively involved in clinical tasks, patient care, or coordination roles. This cultural expectation may contribute to slightly higher physical activity levels among older or more experienced nurses, despite assumptions that younger nurses might be more physically active.

**Table 2:** Distribution of participants based on physical activity level (MET)

Physical activity level	Frequency (f)	Percentage (%)
Low	24	20.5%
Moderate	16	13.7%
High	77	65.8%

**Table 3:** Analysis effect of sociodemographic and physical activity level in nurses

Characteristics	Cross-tabulation			*p*-value	*r*
	Physical Activity Level				
	Low	Moderate	High		
**Unit Room**					
● Emergency Room	16 (28.1%)	9 (15,8%)	32 (56%)	0.196	0,12
● Outpatient Room	8 (13.7%)	7 (7.7%)	45 (27.4%)		
**Gender**					
● Male	8 (22.9%)	5 (14.3%)	22 (62.9%)	0.285	0,10
● Female	16 (6.8%)	11 (4.3%)	55 (67.1%)		
**Age (year)**					
● 26–35	5 (22.7%)	4 (18.2%)	13 (59.1%)	0.006*	0,25
● 36–45	14 (25.5%)	8 (14.5%)	33 (60%)		
● 46–55	5 (17.9%)	3 (10.7%)	20 (71.4%)		
● 56–65	0 (0%)	1 (8.3%)	11 (91.7%)		
**Length of works (year)**					
● 1–3	3 (37.5%)	2 (25%)	3 (37.5%)	0.010*	0,24
● 4–9	1 (9.1%)	2 (18.5%)	8 (72.7%)		
● 10–18	11 (32.4%)	5 (14.7%)	18 (52.9%)		
● 19–21	3 (15.8%)	2 (10.5%)	14 (73.7%)		
● > 21	6 (13.3%)	5 (11.1%)	34 (75.6%)		
**Career level**					
● Clinical Nurse I	5 (31.3%)	3 (18.8%)	8 (50%)	0.060	0,17
● Clinical Nurse II	7 (21.2%)	6 (18.2%)	20 (60.6%)		
● Clinical Nurse III	11 (16.4%)	7 (10.4%)	49 (73.1%)		
● Clinical Nurse IV	1 (100)	0 (0%)	0 (0%)		
● Clinical Nurse V	0 (0%)	0 (0%)	0 (0%)		

Notes: *Significant *p* < 0,050 using Bivariate analysis

## Discussion

This study determined and explored the physical activity level of nurses and the demographic characteristics that might influence it. Our results show that the majority of nurses were categorized as having a high physical activity level, indicated by a MET score greater than 3000 min per week. The finding is in line with another study In Indonesia which found the majority of nurses in the study have higher MET scores [28]. The cause of this finding might be the nurses’ workload that needed to be done quickly and accurately, so the demands of their physical activities are very high [6]. There are also certain factors that affect the overall level of physical activity among nurses, such as unit room, which is discussed in the following paragraph.

### Unit room

This study found that there was no significant correlation between the unit rooms (OR and ER) and their physical activity levels. Even though OR and ER have different responsibilities, hospital factors such as similar nurse-to-patient ratios and standardized task allocation policies may homogenize physical activity levels across units, effectively attenuating differences in physical demands [29]. Previous studies have shown that nurse workload often varies between day and night shifts, depending on the number of patients and available staff [6, 30]. Hospital policies that match staffing to patient numbers may reduce nurses’ need for physical activity. Studies by Needleman et al. (2011) and the AHRQ review (Patient Safety Primer) show that consistent staffing ratios and work assignment policies across units systematically influence the distribution of workload and nursing activities [31].

However, this finding contrast with Philbrick et al. (2022), who reported that nurses in ER have higher activity than those working in other areas [32]. ER nurses are expected to be highly skilled because they handle emergency cases that require quick and accurate responses, whether surgical or non-surgical [33, 34]. Interestingly, ER nurses seem to use pacing strategies, adjusting their efforts during shifts to manage physical fatigue [35]. One study shown that after doing moderate to vigorous activity in one shift, ER nurses showed reduced activity in the following shift, suggesting pacing behavior [36]. On the other hand, nurses in outpatient setting might have different routines, such as more frequent patient visits, which could increase physical activity. This finding is consistent with a study reporting increased physical workload in crowded and noisy service areas, such as the OR [37]. The high workload of nurses affects their attitudes and intentions toward physical activity in their free time, allowing the data to become homogeneous [13]. In addition, individual factors such as age, experience in the unit, and personal attitudes toward physical activity also determine the level of physical activity [6].

### Age

This study shown a weak but statistically significant correlation between age and physical activity level among outpatient and emergency nurses. Most nurses in this study were aged 36–45 years, and physical activity levels tended to be lower in older age groups. This finding aligns with previous studies indicating that physical activity generally declines with age, partly due to age-related changes in muscle mass, physical endurance, and strength [38].

Several studies have supported this pattern, Chang & Cho reported a significant association between younger age and a higher number of steps during a work shift (*p* < 0.001) [6]. Similarly, Philbrick et al. found that nurses aged 18–34 years reported higher total weekly physical activity compared to those aged 35–54 and over 55 years, with the differences reaching statistical significance in the older age group comparisons [32]. McCarthy et al. also reported that nurses aged over 40 years were significantly less likely to meet recommended physical activity levels at work compared to younger nurses [OR 0.47, 95% CI (0.25–0.88), *p* = 0.02] [39].

Although physical activity is often reduced in older adults, the positive correlation found in this study suggests that other factors may contribute to increased activity among older nurses in Indonesia. In this context, older nurses often remain in physically demanding roles due to staff shortages and uneven workforce distribution. National demographic trends, including a growing proportion of older adults, may further contribute to continued workforce participation among older nurses, despite declining physical capacity [40].

Moreover, differences in employment systems may also play a role. For example, in Indonesia, many nurses perform both nursing and non-nursing tasks, with over 90% reporting responsibilities outside core nursing duties. In the context of Indonesian healthcare settings, this may also be amplified by systemic factors such as limited staffing, high patient loads, as minimal support from auxiliary personnel, so all the nurses often undertake more physically demanding tasks. In contrast, nurses in some European countries often receive greater support from auxiliary staff, allowing them to focus more on essential nursing role [39]. For instance, a Finnish study among home care nurses reported that relative physical workload among older and younger nurses were comparable, importantly, there were no significant differences in perceived overall work ability across age groups, suggesting similar duty expectations regardless of age [41]. Thus, the findings of this study indicate that in Indonesia there is a significant relationship between nurses’ physical activity levels and their age.

### Length of works

This study found that length of work has an influence on physical activity levels. One possible explanation is that the shorter the unit experience was significantly associated with a longer distance traveled on all three shifts (*p* < 0.001 for all). This concludes that the higher the degrees are, and the longer the unit experiences, the higher the physical activity level [42]. Nurses with a bachelor’s or higher degree had a higher number of steps on all shifts, and this difference was significant for the night shift (*p* = 0.015) and all shifts (*p* = 0.049) [6].

Contrary to the South Korea study, it stated that nurses with a short tenure have higher physical activity than nurses with a longer tenure. This can be seen from nurses with less than one year of experience in the unit having an average number of steps of 11,000 steps or more, while nurses with three years or more of experience had 7,000 steps. Thus, the least experienced nurses walked about 40% more than nurses who had three or more years of experience. The differences may occur due to differences in competencies and roles according to length of service, such as nurses with less than one year of experience performing more direct nursing care than nurses with longer experience, and possibly a large amount of walking between rooms or due to missing necessary items due to lack of experience [6].

### Career level

The nursing career level is a structured process that assigns nurses to levels based on their knowledge and proficiency, while allowing them to refine their approach to align with their skills. Nurse performance and competency are positively impacted by the nurse career ladder system. In Indonesia, the professional nursing career path comprises four fields: Clinical Nurse, Nurse Manager, Nurse Educator, and Nurse Researcher. Each includes five competency levels. Clinical Nurse I provides basic nursing care under supervision with a focus on technical skills. Clinical Nurse II delivers holistic care independently and manages client groups with guidance for complex cases. Clinical Nurse III offers specialized care, develops evidence-based services, and facilitates clinical learning. Clinical Nurse IV manages complex cases using interdisciplinary approaches, conducts research, and advances clinical education. Clinical Nurse V provides expert consultations, leads transdisciplinary clinical governance, and conducts research to develop nursing practice, education, and the profession [43].

The results of this study showed that there was no significant relationship between career level characteristics and nurses’ physical activity levels. This finding is quite contrasting with previous studies, even though there are no previous studies that mention the specific relationship between career level and the physical activity of nurses. It is known that the likelihood of nurses meeting prescribed amounts of physical exercise at work and in their own time was more than doubled for those with high quantitative demands, meaning that higher career levels may usually experience higher quantitative demands, which increase the amount of physical activity [39]. In Indonesia, nurses’ career levels are not directly linked to physical activity, as advancement is based more on education, experience, competence, and managerial duties than on job physicality. Physical activity is influenced more by task type and work setting than by career rank for instance, a senior ER nurse may be more physically active than a higher-ranking nurse manager. Moreover, according to Minister of Health Regulation No. 26 of 2019, all nurses with a valid registration certificate and practice license are authorized to perform the same duties, regardless of career level.

### Strength and limitation

This study offers several strengths. It addresses an underexplored area by directly comparing physical activity patterns between emergency and outpatient nurses, two groups with distinct occupational demands in high-intensity clinical environments. The analysis incorporated multiple work-related demographic factors: age, gender, length of work, and career level, providing a nuanced understanding of determinants of nurses’ physical activity. The use of standardized metabolic equivalent (MET) scores enhanced measurement precision and facilitated comparability with previous studies. Moreover, the findings have practical relevance for occupational health by identifying subgroups that may benefit from targeted interventions, offering baseline evidence to inform institutional policy and guide future longitudinal or interventional research.

However, its cross-sectional correlational design precludes causal inference, and observed associations may reflect reverse causation or selection bias. Physical activity was self-reported, introducing the potential for recall and social-desirability bias, with limited ability to differentiate between occupational and non-occupational activity domains. The single-institution sample limits generalizability to other nursing populations or healthcare systems with different organizational structures, shift patterns, and cultural norms. Finally, unmeasured factors such as workload intensity, shift type, and access to exercise facilities may have confounded the associations. These limitations should be considered when interpreting the results, and confirmatory studies using longitudinal designs, objective activity monitoring, probability-based sampling, and comprehensive confounder assessment are warranted.

## Conclusion

This study highlighted that most emergency and outpatient nurses reported high levels of physical activity. Significant associations were identified between physical activity levels and both age and length of work, suggesting that these demographic factors may have played a role in influencing nurses’ engagement in physical activity. However, no significant relationships were detected with other demographic variables. Overall, these findings underscored the relevance of incorporating age and work experience into the development of targeted strategies to enhance physical activity among nurses.

## Data Availability

The datasets used and analyzed for the current study are available from the corresponding author upon reasonable request.
